# Influence of Ca_3_(PO_4_)_2_ on the Surface Morphology and Properties of a CaO-Al_2_O_3_-SiO_2_-Fe_2_O_3_-Based High Temperature Phase Reconstructed Complex

**DOI:** 10.3390/molecules29163740

**Published:** 2024-08-07

**Authors:** Huanyin Yang, Hongli Guo, Hongjuan Sun, Tongjiang Peng

**Affiliations:** 1 Key Laboratory of Solid Waste Treatment and Resource Reuse, Ministry of Education, Southwest University of Science and Technology, Mianyang 621010, China; 2 College of Electronic and Information Engineering, Yangtsze Normal University, Chongqing 408100, China; guohongli1211@163.com; 3 Institute of Mineral Materials and Applications, Southwest University of Science and Technology, Mianyang 621010, China; 4 Analysis and Testing Center, Southwest University of Science and Technology, Mianyang 621010, China

**Keywords:** opacity glaze, structural color, phase-separation structure, Ca_3_(PO_4_)_2_, fly ash

## Abstract

In this study, a glaze slurry was prepared with different contents of tricalcium phosphate. It was then applied to a fly ash microcrystalline ceramic billet and sintered at 1180 °C for 30 min to prepare the complex. The aim was to obtain a high value-added application of fly ash in order to reduce environmental pollution. The study systematically investigated the influence of the Ca_3_(PO_4_)_2_ content on the crystal phase evolution, physical-mechanical properties, and micro-morphology of the complex. The results showed that products sintered at 1180 °C with 8 wt% Ca_3_(PO_4_)_2_ demonstrated better performance, with a water absorption of 0.03% and a Vickers microhardness of 6.5 GPa. Additionally, the study observed a strong correlation between the Ca_3_(PO_4_)_2_ content and the opacity effect. A feasible opacity mechanism was also proposed to explain the variation of glaze colors and patterns with different contents of Ca_3_(PO_4_)_2_.

## 1. Introduction

With the rapid development of the economy, various industrial wastes, including fly ash, have emerged. Fly ash contains valuable elements such as iron, silica, alumina, and calcium oxide, making it an attractive resource for recycling and utilization [1,2,3,4,5]. Many studies have addressed the environmental pollution caused by fly ash and explored high-value utilization methods. For instance, researchers have investigated the use of fly ash in the preparation of glass-ceramics [5,6,7,8,9,10]. Due to fly ash’s high Al_2_O_3_ content and low CaO content, additional raw materials are often required to enhance the product performance. Yang et al. utilized fly ash and blast furnace slag as the primary raw materials to create waste slag glass-ceramics through a direct sintering method, studying the impact of fly ash content on the crystalline phase composition and properties of glass-ceramics [11]. Meanwhile, Lu et al. combined glass and fly ash, supplemented with 10% magnesium oxide, to produce glass-ceramics via direct sintering. This resulted in the main crystal phase of pyroxene and magnesia olivine. The product displayed high strength and low density, making it suitable for building materials [12]. However, it is worth noting that the preparation of microcrystalline ceramics solely from fly ash has not yet been reported.

In order to expand the application range of solid waste glass-ceramics, many researchers are exploring various glazes that can be applied to its surface in order to obtain more desirable properties. Research on glaze modifiers has a long history and has produced a wide variety of results. Due to their strong covering ability and decorative effect, colored glazes and crystalline glazes have become the focus of research [13,14,15,16,17,18,19]. Qiu Baixin et al. have prepared an environmentally friendly black ceramic glaze using chromium waste residue for the color precursor system of Fe and Cr elements [20]. This black glaze demonstrates good high temperature color stability, as well as acid and alkali resistance. Wang Yi et al. used CuO and Fe_2_O_3_ as colorants, minerals, and chemical raw materials to successfully prepare red–purple, blue–purple, and other colored glazes, as well as multi-color flower glazes under the same firing atmosphere [21]. The results show that the coloring elements of copper and iron have various coloring properties, and that small changes in process parameters and basic components result in corresponding changes in the color and tone of the glaze. The variety of chemical colors in the glaze, coupled with the micro- and nanostructure, make the glaze coloring phenomenon more complex and changeable, thus increasing the probability of kiln changes and the specificity of color. Numerous studies have shown compound characteristics of chemical color and structural color in the color of ceramic glaze, and the colorant can produce a variety of colors in the ceramic phase separation opaque system [21,22,23,24,25]. The color of a ceramic glaze is a result of liquid immiscibility, creating a phase separation structure in the ceramic with varying refractive indices based on crystal size, resulting in a unique structural color. Xiaotao Shi et al. developed a Tianmu glaze using local raw materials and traditional techniques under alternating oxidation and reduction atmospheres [26]. Testing revealed that the glaze’s color is not only due to iron oxide but also from film interference, coherent diffraction, and small grain or particle scattering. The color rendering of the glaze depends on the atmosphere and the choice of opacification agent. Tricalcium phosphate is chosen as the opacification agent for its cost-effectiveness and environmental suitability, creating a glaze unaffected by atmosphere and suitable for fly ash ceramic billets.

A previous study stated that a glaze with opacity was created by applying the glaze slurry to a ceramic billet that was pressed using pure fly ash from a specific location [27]. The enamel complex was prepared using a single-sinter process. Meanwhile, the influence of the quartz sand content in the glaze on the surface morphology and mechanical properties of the complex glaze layer was studied. The aim of the study was to enhance the surface properties of fly ash microcrystalline ceramics, so as to improve its market competitiveness as a common decorative material, and provide a new way for the high value-added application and sustainable utilization of fly ash. In this paper, based on the previous work, the effects of tricalcium phosphate content on the microstructure and mechanical properties of the glaze layer of the enamel complex were studied, and the opacifying mechanism of the glaze layer was also discussed. This study not only provides theoretical support for enriching the surface color of solid waste ceramics and improving its decorative effect, but also provides technical support for enhancing the mechanical properties of the glaze layer by controlling the formation of the crystal phase.

## 2. Experiment

### 2.1. Sample Preparation

Fly ash from a place in Sichuan was pressed into shape by a tablet press (d = 2 cm), which is used as the glaze substrate. Its chemical composition is shown in Table 1. The raw glaze slurry was prepared using kaolin, potassium feldspar, wollastonite, quartz sand, fired talc, commercial grade calcium phosphate, and zinc oxide. The chemical compositions of these raw materials are given in Table 2. On the basis of the previous work, the raw glaze slurries with different calcium phosphate content were prepared by mixing 5 wt.% kaolin, 45 wt.% potassium feldspar, 14 wt.% wollastonite, 15 wt.% quartz sand, 10 wt.% fired talc, 3 wt.% ZnO (calcined), 0–15 wt.% Ca_3_(PO_4_)_2_, with 0.8 wt.% sodium carboxyl methyl cellulose (CMC) and 0.3 wt.% sodium tripolyphosphate (STPP), according to a material–ball–water ratio of 1:2:0.75 in a ball mill at a rate of 750 r/min for 30 min. Then the raw glaze slurries were applied to the green bodies (d = 2 cm) by brushing. After drying, the samples were sintered at a temperature of 1180 °C for 30 min. Finally, the samples were naturally cooled down to room temperature in the furnace. The sintered samples with different tricalcium phosphate contents, labeled G1–G5, are listed in Table 3.

### 2.2. Characterization

The chemical compositions of the raw materials were determined by X-ray fluorescence spectroscopy (XRF, Axios, Almelo, The Netherlands). The phase and the crystal structure of the sintered samples were measured via X-ray diffraction (XRD, D/MAX-IIB, Tokyo, Japan). The surface morphology of the sintered samples was observed with a digital camera. Microstructural observations of the surface of the sintered samples were conducted using a field emission scanning electron microscope (SEM, JEOL JSM-6390A, Tokyo, Japan) to characterize the morphology and investigate the influence of tricalcium phosphate on the internal glaze microstructure. The composition analysis and element distribution of the crystal structures in the glaze layer were determined through map scanning and point scanning tests using energy-dispersive spectroscopy (EDS).

The water absorption of the sintered samples was determined according to the Archimedes’ principle using the boiling method, and calculated by using the following expression:

(1) $$w=\frac{m_{1}-m_{0}}{m_{0}}\times 100\%$$

where $m_{0},m_{1}$ and w represent the dry weight of the sample in air, the mass of the water-saturated sample in air, and the water absorption, respectively.

A micro-hardness tester (HV-1000A, Laizhou, China) was used to measure the Vickers hardness of the sintered samples under the condition of a load of 500 g applied for 30 s to scratch their surfaces. At least five scratches were made on each sample to obtain reliable data.

## 3. Results and Discussion

### 3.1. Surface Morphology

**Figure 1:**
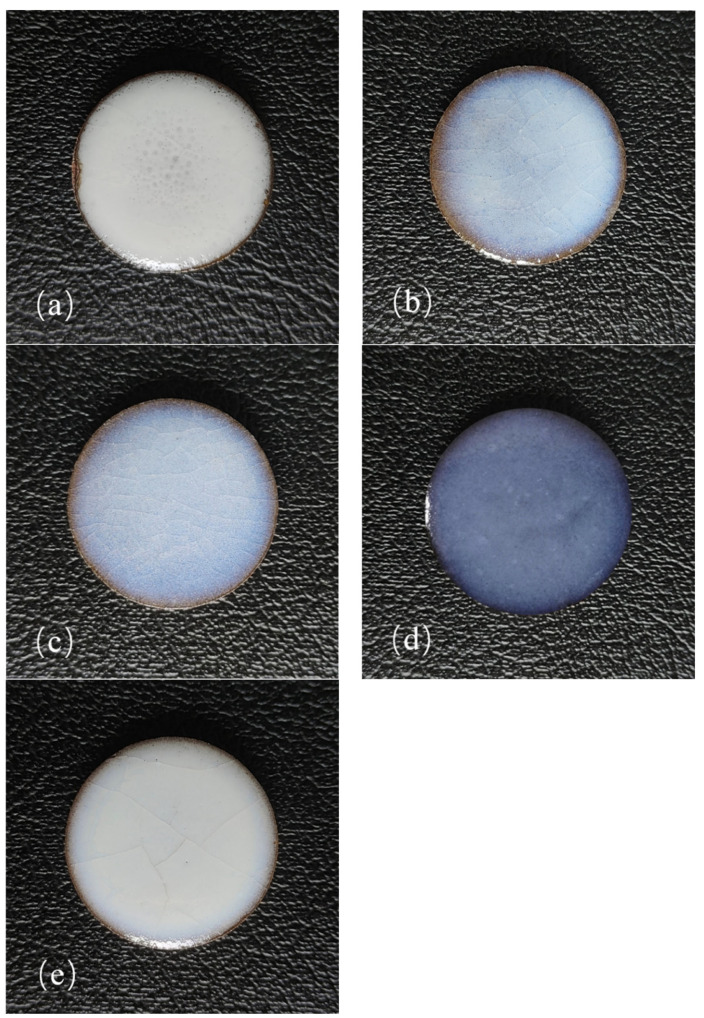
The digital photos of sintered samples. (**a**) G1—0 wt.% TCP, (**b**) G2—4 wt.%, (**c**) G3—6 wt.%, (**d**) G4—8 wt.%, (**e**) G5—10 wt.%.

Figure 1 shows the digital photographs of the sintered samples. Figure 1a–e represents the sintered samples (labeled G1–G5) with a TCP content of 0 wt.%, 4 wt.%, 6 wt.%, 8 wt.%, and 10 wt.% in the glaze paste formula, respectively. It is evident that the glaze layers of samples with added TCP fit flawlessly on the green bodies, exhibiting excellent opalescence and strong covering ability. The reason for this may be that the thermal expansion coefficient of the glaze layer without TCP is very different from that of the billet, and then the phenomenon of rolling glaze or delamination occurs in sample G1. While the addition of tricalcium phosphate may change the thermal expansion coefficient of the glaze layer, making it closer to the thermal expansion coefficient of the body, it can be firmly combined with the body [28,29]. In addition, the glaze layer of sample G1 (0% TCP) shows a significant number of holes. This may be due to organic matter, moisture, or gases not being fully expelled during the sintering process, leading to the formation of these holes. Additionally, the presence of more crystal phases in G1 and less glass phases, as indicated by the XRD results, causes disorganized crystals to pile together. This results in poor fluidity of the molten glass body and the inability of the glaze to fill the gaps, creating many holes in the glaze layer, as observed in the SEM images. However, these crystals and pores of a certain size, shape under the use of light scattering, interference, and other phenomena, can create a certain opacification effect, so it also shows a good covering ability.

Samples G2–G5 show no noticeable holes in the glaze layers. This is due to the addition of TCP, which reduces the viscosity and increases the fluidity of the glaze. As a result, the glaze can discharge gas more easily and form a more continuous and dense glass phase, filling numerous voids. Stress cracks are evident in samples G2, G3, and G5, becoming more pronounced with increasing TCP dosage. The reason is that TCP reacts with other glaze components to form new compounds, altering the microstructure and impacting the elastic modulus of the glaze layer. For example, tricalcium phosphate can be decomposed into calcium oxide at high temperature, which forms diopside crystals with silicon, magnesium, and other elements in the glaze, which can be reflected on the XRD pattern. It can also be verified by XRD patterns that tricalcium phosphate may also react with magnesium oxide in fired talc to form whitlockite (magnesian), which can improve the elastic modulus of glaze. It can be seen from the EDS element distribution mapping that P, Al, and other elements are evenly distributed without obvious aggregation or separation phenomenon. It can be inferred that tricalcium phosphate may form an aluminum–phosphate solid solution with alumina in the glaze containing Al_2_O_3_, which may also improve the elastic modulus. If the elastic modulus increases, the glaze layer’s stress adaptability decreases, raising the risk of stress cracking. Furthermore, the thermal expansion coefficient of the glaze layer changes when TCP is added, affecting the thermal compatibility between the glaze layer and the billet. Insufficient or excessive amounts can cause a mismatch in the coefficient of thermal expansion, leading to thermal stress during cooling and the formation of stress cracks [30,31]. Sample G4 exhibits a good blue opacification effect, and the glaze is smooth and free from obvious defects, resulting in a beautiful effect.

### 3.2. Phase Analysis

**Figure 2:**
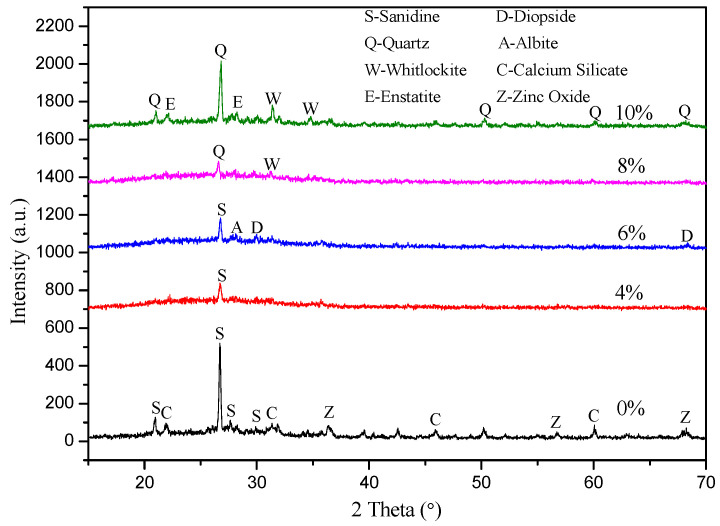
XRD patterns of glaze surface of samples with different TCP contents.

Figure 2 shows the XRD patterns of glaze surface of samples with different TCP contents (G1–G5). Clearly, in sample G1 without tricalcium phosphate, the main phase detected is sanidine (high, syn, PDF #00-010-0353, KAlSi_3_O_8_). In addition, small amounts of calcium silicate (PDF #00-003-1068, CaSiO_3_) and zinc oxide (PDF #01-075-0576, ZnO) are also present. With the amount of tricalcium phosphate increasing, the peak of sanidine decreases progressively, and the peaks for calcium silicate and zinc oxide also disappear. However, it can also be observed that the faint albite (heat-treated, PDF #01-089-6427, Na (AlSi_3_O_8_)) and diopside (PDF #01-075-0945, CaMg(SiO_3_)_2_) peaks are present in sample G3, having 6 wt.% of added TCP. It is clear that there is no significant sanidine peak detected in sample G4, having 8 wt.% of TCP. However, peaks for quartz (PDF #01-085-1054, 00-002-0471, SiO_2_) and whitlockite (PDF #00-015-0389, 00-003-0713) are beginning to appear, and their intensities increase notably with higher TCP content. Additionally, a faint peak of enstatite (ferroan, PDF #00-019-0606, MgSiO_3_) is also identified in sample G5 (10 wt.% TCP). Upon careful observation, a broad hump in the 2θ range between 15° and 35° indicates the formation of a glass phase during the sintering process [32]. The bulge in the glass phase peak gradually increases with TCP addition and then decreases when the TCP content exceeds 8 wt.%.

The peaks of samples G1 (0wt.% TCP) and G5 (10wt.% TCP) are not as prominent because they contain more phases. The effect of TCP on the structure of the glaze suggests that the formation of the glass phase is initially enhanced. As the tricalcium phosphate content increases, the crystal phase is precipitated, inhibiting the further formation of the glass phase. This phenomenon can be explained by the fact that TCP with a lower content can enter the silicate network structure as a network modifier and reduce the polymerization degree of the network by reacting with the [SiO_4_] tetrahedron, thus promoting the formation of the glass phase [33]. The addition of TCP helps to reduce the viscosity of the glaze and increase its fluidity, resulting in the formation of a more continuous and dense glass phase during the sintering process. During the high-temperature sintering process, TCP breaks down and releases phosphate ions (PO_4_^3−^) and calcium ions (Ca^2+^). These ions act as crystal nuclei and react with other components in the glaze, such as SiO_2_ and Al_2_O_3_, to encourage the formation of new crystal phases. For instance, Ca^2+^ can combine with [SiO_4_] tetrahedrons in silicate networks to create calcium-containing silicate minerals like diopside. When the TCP content reaches and exceeds 8wt.%, tricalcium phosphate starts to precipitate as independent crystal phases, including quartz and whitlockite. This process reduces the number of glass phases in the disordered network structure, thus enhancing the hardness of the glaze layer and improving its mechanical strength, while also creating an opacification effect.

### 3.3. Physical and Mechanical Properties

**Figure 3:**
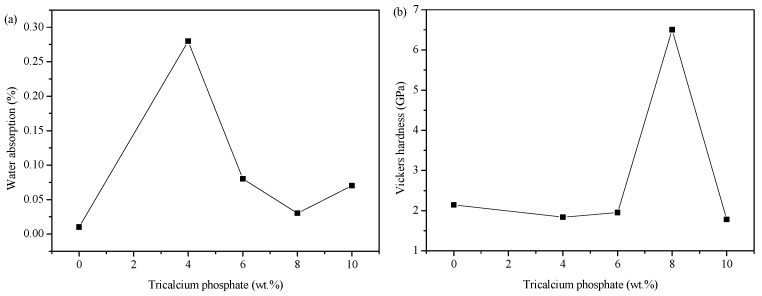
(**a**) Water absorption, (**b**) Vickers hardness of samples G1–G5.

The influence of the TCP content on the physical and mechanical properties of the samples is shown in Figure 3a,b, which demonstrate that the TCP content reaches a turning point at 8 wt.%.

In Figure 3a, it is observed that the water absorption of the samples labeled G2–G5 initially decreases, then increases with the rise in TCP content. The lowest values are seen at 8 wt.%. Additionally, it is evident that the water absorption of sample G1 is lower than that of the TCP-added samples. This may be due to the fact that the glaze of sample G1 has better waterproofing properties owing to its dense vitreous body. On the other hand, the vitreous body of the samples with added TCP is less dense, with some samples having more cracks, resulting in higher water absorption. When the TCP content increases, more and more of the molten body fills some of the pores in the glaze, leading to decreased water absorption. However, upon reaching a 10 wt.% TCP content, the crystal phase content in the glaze increases, while the glass melt decreases, causing an increase in glaze porosity and the appearance of stress-related cracks, leading to increased water absorption in sample G5. In Figure 3b, it is shown that the Vickers hardness of sample G4 increases significantly, while the others do not change notably. However, among the samples other than G4, sample G1 without TCP exhibits slightly higher Vickers hardness. The Vickers hardness of the sample decreases initially with the addition and increase in content of TCP, then it increases, decreases again, and reaches its maximum value at 8 wt.%. This suggests that adding TCP has a significant impact on the glaze performance. The type of crystal phases formed during the high temperature phase reconstruction in the glaze layer is influenced by the chemical constitution and the high temperature viscosity of the glaze, while also being affected by the process conditions [34,35]. In the glaze with a lower TCP content, the main crystalline phase is sanidine, which has a higher Mohs hardness and can improve the hardness of the glaze layer. Sample G1 has a higher hardness due to the high content of sanidine crystals and the dense vitreous body in the glaze layer. As TCP is added, the hardness of the glaze layer gradually decreases due to the decrease in crystal content caused by dissolution and the increase in vitreous body content. At 8 wt.%, the hardness improves as quartz crystal with higher hardness begins to precipitate. However, at 10 wt.%, stress defect cracks appear and reduce the hardness of the glaze layer, despite the increased quartz crystal content. 

In summary, the physical and mechanical properties of sample G4 (8 wt.%TCP) are better, its water absorption rate is 0.03%, Vickers hardness is 6.5 GPa, and it is a product with high water resistance and high wear resistance among glazed ceramic materials [22,36,37].

### 3.4. Microstructure and EDS Analysis

**Figure 4:**
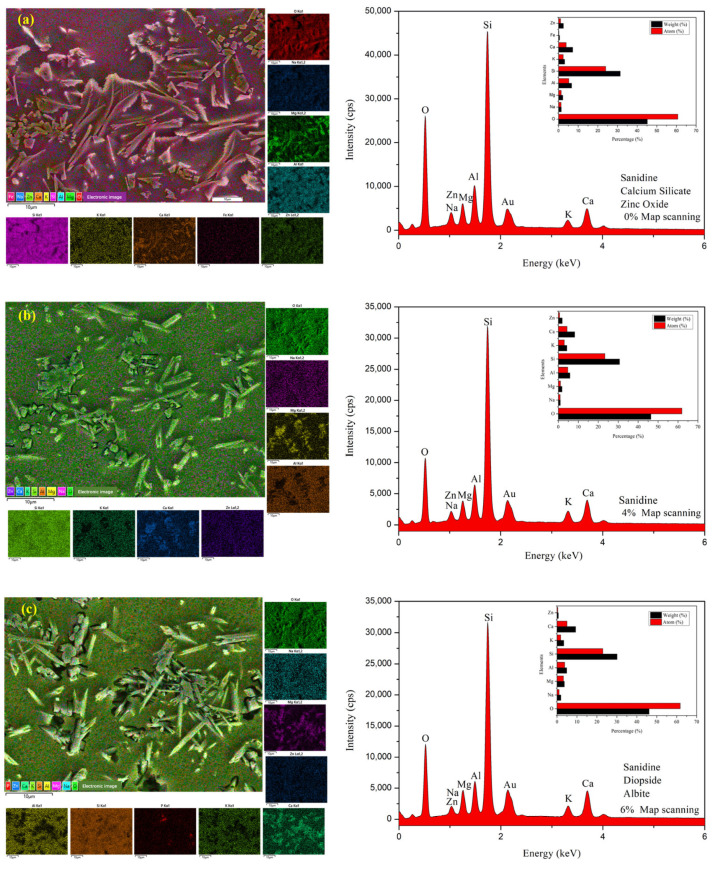

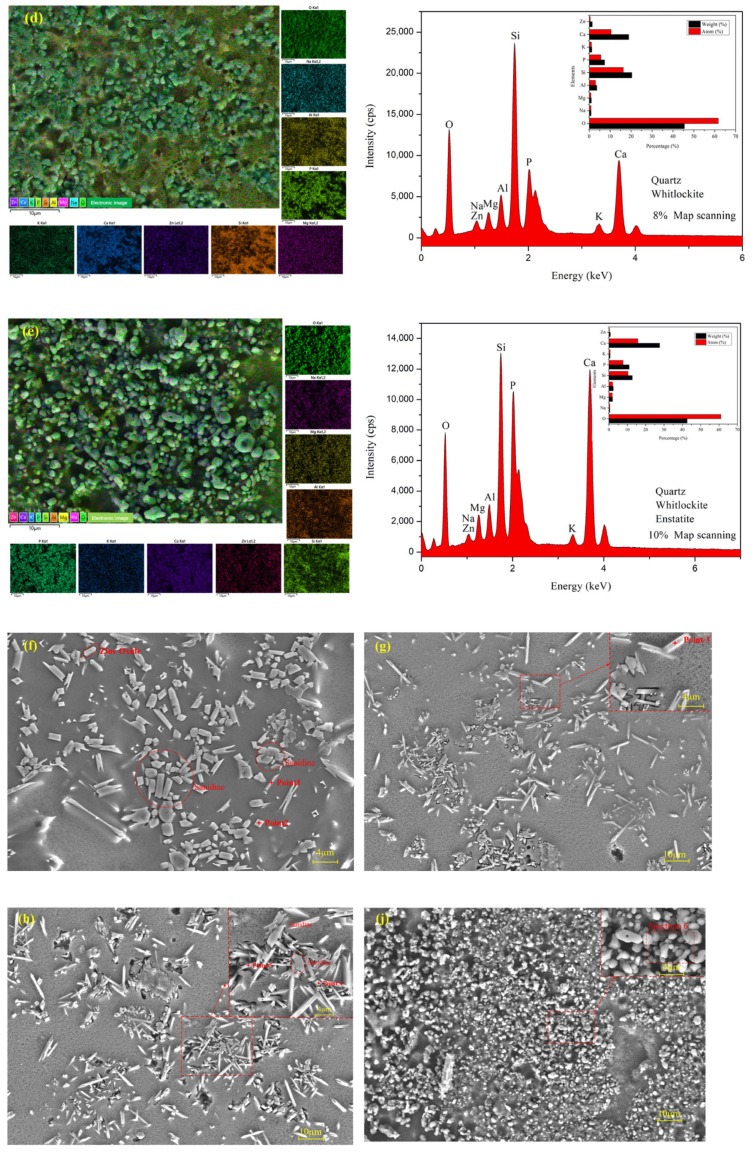

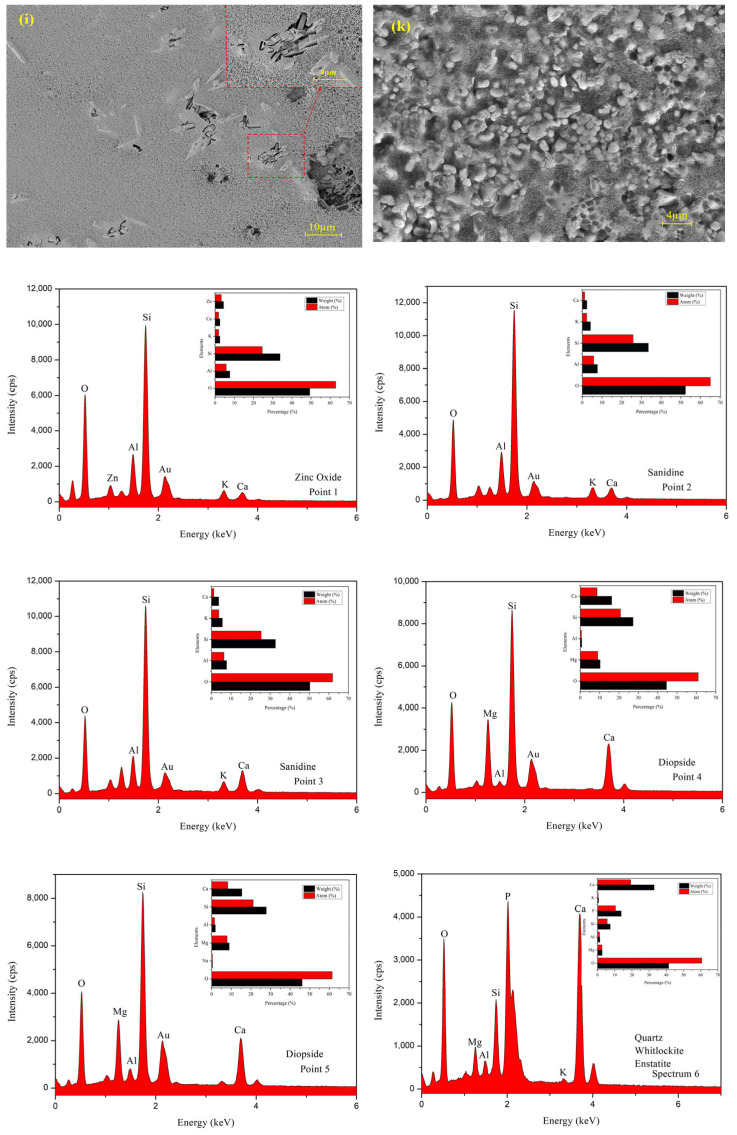
The SEM micrographs and EDS spectra on the glaze surfaces of the samples for G1–G5. (**a**–**e**) present the elemental mapping images and map scanning spectra on part of the glaze surfaces of the samples for G1–G5, which have TCP contents of 0 wt.%, 4 wt.%, 6 wt.%, 8 wt.%, and 10 wt.%. (**f**–**k**) show the SEM images of the crystals and EDS spectra of the points inside the red dotted box on the glaze surfaces of samples G1–G5.

Figure 4 illustrates the micromorphologies and EDS spectra of the glazed surface of all samples sintered at 1180 °C for 30 min. The samples all contain a large number of irregularly shaped crystals distributed within a glass matrix. In sample G1, Figure 4a,f illustrate the presence of various crystal structures with massive columnar, rod-like, rhombohedral, and granular shapes. The XRD analysis in Figure 2 and the crystal morphologies suggest the presence of sanidine, calcium silicate, and zinc oxide. The chemical compositions of specific crystal groups and two representative crystals labeled as point 1 and point 2 were analyzed using EDS map scanning and dot scanning in Figure 4a,f. The analysis revealed that the crystal at point 1 is rich in O, Al, Si, and Zn, indicating it is likely zinc oxide. The crystal at point 2 was found to be rich in O, Al, Si, K, and Ca, suggesting it is sanidine. The presence of three types of crystal structures in sample G1 was confirmed through map scanning energy spectrum and atomic proportion analysis. It is possible to verify the crystal structures in other samples using SEM morphologies and EDS analysis, combined with XRD results, as shown in Figure 4b–e,g–j.

Figure 4f shows that most of the crystals in the glaze layer of sample G1 are exposed on the surface, and they are short and thick in size. The vitreous body is relatively dense, and the encapsulation between the vitreous body and the crystal is poor. It is speculated that the glaze, under this formula, has high viscosity, poor fluidity, and a smaller molten volume. There are gaps in the crystals due to piling up together, which cannot be completely filled by the molten body, resulting in obvious pores in the glaze layer. With the addition of TCP, the Ca^2+^ reacts with other materials in the glaze, resulting in the destruction of the silicon–oxygen tetrahedral network structures. This can reduce the melting temperature of the glaze and decrease the viscosity. As a result, the fluidity of the glaze is enhanced, and the molten body can fill the gaps created by the accumulation of crystals to better enclose the crystals (as can be seen from Figure 4g–k, where the crystals are embedded in the vitreous body and exposed after etching with HF acid). Additionally, the vitreous structure becomes loose, so the hardness of the glaze will also decrease (consistent with the hardness test results). Crystal growth is a dynamic process that is influenced by the dissolution–precipitation mechanism. The weak intermolecular force in the low viscosity glaze allows the crystalline raw material to be more easily dissolved in the glaze. This increased dissolution rate leads to quicker supersaturation of the solution, causing ions or molecules to aggregate and form crystalline nuclei. Additionally, the low viscosity aids the migration and diffusion of ions or molecules in the melt, thus promoting crystal growth. Furthermore, the low viscosity glaze may form more vitreous bodies during the cooling process, wrapping the crystals in these bodies and limiting their growth, resulting in smaller or elongated crystals. In this environment, different crystals may grow simultaneously and compete with each other, and any change in the chemical composition of the glaze will alter the type of crystal phase. The analysis using SEM and EDS indicated that as the amount of TCP increases, the crystal size becomes more slender, and the content of sanidine, the main crystal phase, gradually decreases and eventually disappears. When the TCP content reaches 8 wt.%, quartz becomes the main crystal phase, with a certain amount of whitlockite crystal precipitating as well. This could be due to the fluxing action of TCP, which reduces the melting temperature of the glaze, facilitating the precipitation of quartz crystal. The phosphate ions from the TCP increase the phosphate content in the glaze, leading to the precipitation of whitlockite crystals. The hardness of quartz is higher than that of sanidine, suggesting that the precipitation of quartz crystals may effectively increase the hardness of the glaze layer, as confirmed by the change in hardness values on the mechanical properties curve (Figure 3b).

### 3.5. Opacification Mechanism

**Figure 5:**
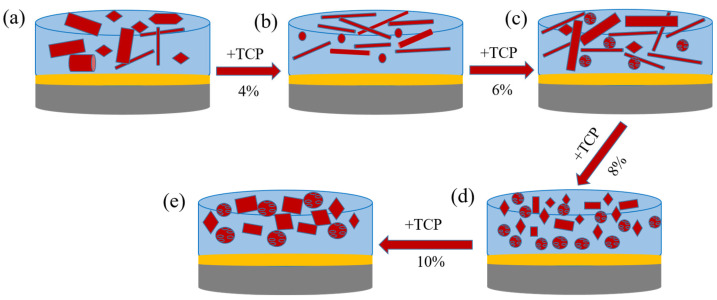
Structural evolution diagram of glaze microstructure with different tricalcium phosphate contents. (**a**) G1, (**b**) G2, (**c**) G3, (**d**) G4, (**e**) G5.

In Figure 5, the evolution of the glaze microstructure with different tricalcium phosphate contents is illustrated, demonstrating the mechanism of glaze opacification. It is well known that the structural color is influenced by the internal microstructure of the glaze layer. By adjusting the glaze formula, the glaze layer can undergo liquid–liquid phase separation or develop microbubbles, microcrystalline structure, etc. This selective manipulation allows the glaze layer to reflect, scatter, transmit, or diffract natural light, producing different colors without the need for coloring materials. This environmentally friendly and healthier approach utilizes the structural color of the glaze layer, following the opacification mechanism of phase separation and molten crystallization. This results in Rayleigh scattering and Mie scattering phenomena of light generated by the phase-separated structure and the microcrystals in the glaze layer. The blue opalescence comes from the former, while the latter appears as milk white. Rayleigh scattering explains the scattering of particles that are out of phase (such as phase-separated structures and microcrystals) and are smaller in size or close to the wavelength of the light wave. This means it explains the scattering of smaller particles. For larger scatterers, Mie scattering is typically used to provide an explanation.

The color mechanism of the glaze layer in this study is as follows: The glaze layer without TCP has been certified with XRD analysis (Figure 2). The analysis indicates that this glaze layer contains fewer non-crystalline glass phases and more crystals. The opacification of the glaze layer is primarily caused by the melted crystals in the glaze. The crystals in the glaze are large and evenly distributed, resulting in Mie scattering, which gives the glaze a white appearance (see Figure 5a). In the glaze melt, the electric field strength of P^5+^ is 2.1, and Si^4+^ is 1.57 [38]. When TCP is added, the high field strength of P^5+^ allows it to compete more effectively for free oxygen compared to Si^4+^. Adding Si^4+^ to the glaze melt increases the incompatibility of the glaze system, leading to the formation of a phase-separated structure consisting of a Si-rich phase and a P-rich cationic phase.

When TCP is added and Ca^2+^ reacts, the viscosity of the glaze melt decreases, resulting in an increased glass phase and decreased microcrystalline phases. When the TCP content is between 4 wt.% and 6 wt.%, some crystals become longer and thinner with an unbalanced distribution, leading to a light blue appearance on the glaze surface due to Rayleigh and Mie scattering (as seen in Figure 1b,c). The results from the EDS map scanning spectrum show that there is a few spherical phase-separated structures in the glaze with a small phosphorus content, and where the melted crystals play a main role in the opacity of the glaze layer. When the TCP content reaches 8 wt.%, we observe through the SEM (Figure 4k)/EDS map scanning spectrum (Figure 4d) and XRD analysis that there are molten crystals and spherical phase-separated structures present in the glaze layer. These structures range in size from tens of nanometers to 1 micron, which is similar to the wavelength of visible light, allowing for Rayleigh scattering and resulting in a good blue opacification effect. When the content increases to 10 wt.%, according to the XRD analysis, the amorphous glass phase of the glaze decreases, the crystal content increases, and the crystal size increases to between several hundred nanometers and 3 microns. This size is greater than the incident light wavelength, so most of them comply with Mie scattering. As a result, the glaze appears white.

## 4. Conclusions

The glaze slurry prepared with different contents of tricalcium phosphate was applied to pure fly ash base and sintered at 1180 °C for 30 min to prepare the complex. When the content of the tricalcium phosphate is 8 wt.%, the glaze layer of the sample has a smooth blue opacification effect without obvious defects. At this time, the water absorption of the sample is 0.03%, which is significantly lower than the standard of a glazed tile, indicating that the sample is very waterproof. Meanwhile, the Vickers microhardness of the sample glaze layer is 6.5 GPa, which is a high hardness value in the glazed tile, indicating that it has excellent wear resistance. These are closely related to the microstructure formed in the glaze layer of the sample during high temperature sintering, and the formation of the microstructure is related to the TCP content. Based on the XRD and SEM/EDS analysis, a mechanism of opacification involving phase separation and molten crystallization is proposed. This mechanism produces different structural colors through Rayleigh scattering and Mie scattering. The structural color is directly related to the microstructure in the glaze layer, which is determined by the TCP content in this study. In glazes without TCP and with a TCP content of 4–6 wt.%, the phase separation is not very obvious, mainly due to the opacification of the microcrystalline phase that has precipitated in the glaze. However, when the TCP content reaches 8%, the phase separation becomes more pronounced, and the opacification is mainly caused by the phase-separated structure and the microcrystalline phase.

## Figures and Tables

**Table 1:** Chemical analysis determined by XRF of industrial waste: fly ashes (FA) (wt.%).

SiO_2_	Al_2_O_3_	CaO	MgO	K_2_O	Na_2_O	ZnO	Fe_2_O_3_	TiO_2_	BaO	ZrO_2_	P_2_O_5_	MnO	SrO	Others	SO_3_
39.13	16.74	7.70	0.33	2.91	0.28	0.07	22.97	5.25	0.16	0.33	0.44	0.17	0.26	—	3.01

**Table 2:** Chemical analysis determined by XRF of glaze raw materials (wt.%).

	SiO_2_	Al_2_O_3_	CaO	MgO	K_2_O	Na_2_O	ZnO	Fe_2_O_3_	TiO_2_	BaO	ZrO_2_	P_2_O_5_	MnO	SrO	Others	SO_3_
Kaoline	54.82	38.19	1.38	—	0.30	0.19	0.01	1.31	3.16	—	0.21	—	—	0.04	0.39	—
Feldspar	62.08	10.54	1.13	—	22.10	0.31	0.02	1.05	—	1.07	0.23	0.12	0.10	0.24	1.01	—
Fired Talc	81.96	0.29	5.75	10.21	—	0.11	—	0.69	—	—	—	0.48	—	—	0.51	—
wollastonite	51.75	—	48.25	—	—	—	—	—	—	—	—	—	—	—	—	—
Quartz	99.21	0.24	0.12	0.06	0.07	0.10	—	0.05	—	0.02	0.00	—	—	—	0.19	0.02

**Table 3:** The weight ratio (wt.%) of tricalcium phosphate (TCP) in different samples.

	Trialcium Phosphate	Other Raw Materials
G1	0	same
G2	4	same
G3	6	same
G4	8	same
G5	10	same

## Data Availability

The original contributions presented in the study are included in the article, further inquiries can be directed to the corresponding authors.

## References

[1] Fernandes H.R., Tulyaganov D.U., Ferreira J.M.F. (2009). Preparation and Characterization of Foams from Sheet Glass and Fly Ash Using Carbonates as Foaming Agents. Ceram. Int..

[2] Yan M., Wen X., Sun Y., Zhou Z., Jiang J., Hu R., Zhang Y., Hantoko D. (2023). Fly Ash Treatment by Desalination Coupled with Solidification of Heavy Metals. Process Saf. Environ. Prot..

[3] Iyer R.S., Scott J.A. (2001). Power Station Fly Ash—A Review of Value-Added Utilization Outside of the Construction Industry. Resour. Conserv. Recycl..

[4] Zhao Y.L., Ye J.W., Lu X.B., Liu M.G., Lin Y., Gong W.T., Ning G.L. (2010). Preparation of Sintered Foam Materials by Alkali-Activated Coal Fly Ash. J. Hazard. Mater..

[5] Zhu M.G., Ji R., Li Z.M., Wang H., Liu L.L., Zhang Z.T. (2016). Preparation of Glass Ceramic Foams for Thermal Insulation Applications from Coal Fly Ash and Waste Glass. Constr. Build. Mater..

[6] Guo Y.X., Zhang Y.H., Huang H.W., Hu P. (2014). Effect of Heat Treatment Process on the Preparation of Foamed Glass Ceramic from Red Mud and Fly Ash. Appl. Mech. Mater..

[7] Barbieri L., Lancellotti I., Manfredini T., Queralt I., Rincon J.M., Romero M. (1999). Design, Obtainment, and Properties of Glasses and Glass–Ceramics from Coal Fly Ash. Fuel.

[8] Albertini A.V.P., Silva J.L., Freire V.N., Santos R.P., Martins J.L., Cavada B.S., Cadena P.G., Rolim Neto P.J., Pimentel M.C.B., Martinez C.R. (2013). Immobilized Invertase Studies on Glass–Ceramic Support from Coal Fly Ashes. Chem. Eng. J..

[9] Wang S.M., Zhang C.X., Chen J.D. (2014). Utilization of Coal Fly Ash for the Production of Glass-Ceramics with Unique Performances: A Brief Review. J. Mater. Sci. Technol..

[10] Li B.W., Deng L.B., Zhang X.F., Jia X.L. (2013). Structure and Performance of Glass–Ceramics Obtained by Bayan Obo Tailing and Fly Ash. J. Non Cryst. Solids.

[11] Yang S.M., Zhang W. (2015). Research on Glass Ceramics of Multi-Solid Waste Slag by Blast Furnace Slag and Fly Ash. Bull. Chin. Ceram. Soc..

[12] Lu Z.Y., Lu J.S., Li X.B., Shao G.Q. (2016). Effect of MgO Addition on Sinterability, Crystallization Kinetics, and Flexural Strength of Glass–Ceramics from Waste Materials. Ceram. Int..

[13] Zhao X.Z. (2016). Review and Prospect of Modern Crystalline Glazes in China. Shandong Ceram..

[14] Pekkan K., Tasci E., Gun Y. (2015). The Effect of ZnO on Development of Crystals in Crystal Glaze Applications. J. Fac. Eng. Archit. Gazi Univ..

[15] Liu P.D., Yu P.L. (2002). Crystalline-Glaze Series of Calcium-Magnesium Silicate—Possible Minerals of Calcium-Magnesium Silicate in Crystalline-Glaze. Ceram. Hanyang.

[16] Chen C.M., Han X.W., Zhao Y.H. (2011). The Study of Tea Dust Crystalline Glaze on Non-Ferrous. China Ceram..

[17] Jamaludin A.R., Kasim S.R., Ahmad Z.A. (2010). The Effect of CaCo_3_ Addition on the Crystallization Behavior of ZnO Crystal Glaze Fired at Different Gloss Firing and Crystallization Temperatures. Sci. Sinter..

[18] Li M., Li W.D., Lu X.K. (2020). Controllable Preparation and Decorative Effect of Iron-Based Crystalline Glazes. J. Chin. Ceram. Soc..

[19] Gajek M., Partyka J., Leśniak M., Rapacz-Kmita A., Wojcik L. (2018). Gahnite White Colour Glazes in ZnO–R_2_O–RO–Al_2_O_3_–SiO_2_ System. Ceram. Int..

[20] Qiu B.X., Gu X.Y., Dong W.X., Luo T., He M.K., Wu P.Y. (2019). Preparation of Black Glaze Using Ferrochromium Slag Waste. J. Chin. Ceram. Soc..

[21] Wang Y., Yu S.H. (2018). Coloring Characteristics and Fambe Mechanism of Iron and Copper Coloring Glaze. J. Chin. Ceram. Soc..

[22] Zhu J.F., Shi P., Wang F., Zhao T., Jiang H. (2016). Preparation of Separative-Phase Fancy Glaze Derived from Iron Ore Slag. Ceram. Int..

[23] Shi P., Wang F., Zhu J.F., Yang H.B., Wang Y., Fang Y., Zhang B., Wang J.H. (2018). Amorphous Photonic Crystals and Structural Colors in the Phase Separation Glaze. J. Eur. Ceram. Soc..

[24] Shi P., Jin Z.W., Wang F., Luo H.J., Zhu J.F., Yang C.G., Liu M., Chen K.Y., Zhang B. (2023). Preparation of a Highly Color-Saturated Amorphous Photonic Crystal Structural Color Glaze by Imitating the Multilayered Structure of Jun Porcelain. Ceram. Int..

[25] Zhang B., He X.L., Ning H.J., Wang F., Zhu J.F., Luo H.J., Fei G.Q., Takeoka Y., Shi P. (2024). Study on the Coupling Effect of Chemical Color and Structural Color for Lushan Speckle Porcelain Glaze in the Tang Dynasty (618–907AD). J. Eur. Ceram. Soc..

[26] Shi X.T., Yu Y.G., Sun Q., Zhu W.Y., Peng C., Wu J.Q. (2019). Mechanisms of Pattern and Colour Generation of Chinese Tianmu Glaze. RSC Adv..

[27] Yang H.Y., Guo H.L., Sun H.J., Peng T.J. (2023). Surface Modification Study of CaO-Al_2_O_3_-SiO_2_-Fe_2_O_3_ Base System High Temperature Phase Reconstruction. Front. Mater..

[28] Capoglu A. (2005). Elimination of Discolouration in Reformulated Bone China Bodies. J. Eur. Ceram. Soc..

[29] Plesingerova B., Klapac M., Kovalcikova M. (2002). Moisture Expansion of Porous Biscuit Bodies—Reason of Glaze Cracking. Ceram. Silik..

[30] Wu J.Q. (2008). The Effect of CaF_2_ and Ca_3_(PO_4_)_2_ on Property of Barium Calcium Silicate Glass. Chin. Ceram..

[31] Chen T.T., Gong B., Tang C.A. (2023). Origin and Evolution of Cracks in the Glaze Surface of a Ceramic during the Cooling Process. Materials.

[32] Zhang C.S., Wang X., Zhu H.J., Wu Q.S., Hu Z.C., Feng Z.Z., Jia Z. (2020). Preparation and Properties of Foam Ceramic from Nickel Slag and Waste Glass Powder. Ceram. Int..

[33] Hui T., Sun H.J., Peng T.J., Liu L., Ding W.J., Wang C., Liu B. (2022). Recycling of Extracted Titanium Slag and Gold Tailings for Preparation of Self-Glazed Ceramic Foams. Ceram. Int..

[34] Wang S.L., Bao Z.H., Miao L.F. (2023). Effect of MgO on Crystallization and Properties of Anorthite Glass-Ceramic Glaze. J. Ceram..

[35] Chi Z.X. (2015). Ceramic Glaze Colorants and Decoration.

[36] de Souza-Dal Bó G.C., Dal Bó M., Bernardin A.M. (2021). Reuse of Laminated Glass Waste in The Manufacture of Ceramic Frits and Glazes. Mater. Chem. Phys..

[37] Gol F., Yilmaz A., Kacar E., Simsek S., Sarıtas Z.G., Ture C., Arslan M., Bekmezci M., Burhan H., Sen F. (2021). Reuse of Glass Waste in The Manufacture of Ceramic Tableware Glazes. Ceram. Int..

[38] Yang C.A. (2016). Research on Separative-Phase Glaze and Its Structural Color. Ph.D. Dissertation.

